# Polyphenol-Rich Extracts Obtained from Winemaking Waste Streams as Natural Ingredients with Cosmeceutical Potential

**DOI:** 10.3390/antiox8090355

**Published:** 2019-09-01

**Authors:** Melanie S. Matos, Rut Romero-Díez, Ana Álvarez, M. R. Bronze, Soraya Rodríguez-Rojo, Rafael B. Mato, M. J. Cocero, Ana A. Matias

**Affiliations:** 1 Nutraceuticals & Bioactives Process Technology Group, Instituto de Biologia Experimental e Tecnológica (iBET), Av. República, Qta. Do Marquês, Estação Agronómica Nacional, Edifício iBET/ITQB, 2780-157 Oeiras, Portugal; 2 BioEcoUVa, Research Institute on Bioeconomy, High Pressure Processes Group, Department of Chemical Engineering and Environmental Technology, School of Engineering, University of Valladolid (UVa), Sede Mergelina Valladolid, 47011 Castilla y León, Spain; 3 Instituto de Tecnologia Química e Biológica António Xavier (ITQB), Universidade Nova de Lisboa, Av. da República, 2780-157 Oeiras, Portugal; 4 Faculty of Pharmacy, University of Lisbon (FFUL), Av. Prof. Gama Pinto, 1649-003 Lisbon, Portugal

**Keywords:** phenolics, antioxidants, anti-ageing, skin whitening, grape marc, wine lees

## Abstract

Phenolics present in grapes have been explored as cosmeceutical principles, due to their antioxidant activity and ability to inhibit enzymes relevant for skin ageing. The winemaking process generates large amounts of waste, and the recovery of bioactive compounds from residues and their further incorporation in cosmetics represents a promising market opportunity for wine producers and may contribute to a sustainable development of the sector. The extracts obtained from grape marc and wine lees, using solid–liquid (SL) extraction with and without microwave (MW) pretreatment of the raw material, were characterized in terms of antioxidant activity through chemical (ORAC/HOSC/HORAC) and cell-based (keratinocytes—HaCaT; fibroblasts—HFF) assays. Furthermore, their inhibitory capacity towards specific enzymes involved in skin ageing (elastase; MMP-1; tyrosinase) was evaluated. The total phenolic and anthocyanin contents were determined by colorimetric assays, and HPLC–DAD–MS/MS was performed to identify the main compounds. The MW pretreatment prior to conventional SL extraction led to overall better outcomes. The red wine lees extracts presented the highest phenolic content (3 to 6-fold higher than grape marc extracts) and exhibited the highest antioxidant capacity, being also the most effective inhibitors of elastase, MMP-1 and tyrosinase. The results support that winemaking waste streams are valuable sources of natural ingredients with the potential for cosmeceutical applications.

## 1. Introduction

Europe is responsible for the largest share of wine production globally, accounting for more than 60% of the world’s entire production [1]. The winemaking process generates large amounts of solid organic waste and by-products, including grape marc (62%), wine lees (14%), grape stalk (12%), and dewatered sludge (12%) [2]. It is estimated that 14.5 million tons of byproducts from wineries are generated annually in Europe alone [3], and the discarding of these dregs may potentially cause environmental issues, due to a low pH and the presence of phytotoxic and antibacterial phenolic substances resisting biological degradation [4]. Grape and wine (poly)phenols are already exploited as cosmetic ingredients due to their renowned antioxidant activities. However, the exploitation of winery wastes is not common yet. In this regard, and since waste streams from the vinification process may present an environmental hazard, the recovery of high-added value bioactive compounds, such as (poly)phenols, from winemaking residues seems a promising market opportunity for wine producers and may contribute to a sustainable development of the sector.

Aged skin is known to have a compromised barrier function, resulting in a dry appearance and susceptibility to environmental aggressors, and therefore is an enhanced risk for skin disorders [5]. Apart from the uneven pigmentation of the epidermis and due to alterations in tyrosinase activity amongst melanocytes [6], the main changes in ageing skin occur at the level of the dermal connective tissue and are essentially translated into the loss of mature collagen and alterations in the elastic network [7]. Among extracellular matrix (ECM)-degrading enzymes, matrix metalloproteinases (MMPs) and elastolytic enzymes (elastases) can be found. These endopeptidases are responsible for the turnover of several ECM components, including all the types of collagen and elastin, playing important roles in numerous physiological processes, such as tissue repair and remodeling, cell migration and differentiation, or wound healing [8,9,10]. However, an exacerbated amount of these enzymes in their active form is the driving cause of several pathological conditions, including accelerated skin ageing.

Reactive oxygen species (ROS), resulting from electron leakage during aerobic metabolism and upon exposure to environmental factors, are unstable species capable of inducing damage to several biomolecules, leading to altered functionality. Hence, to counteract their effect, there are natural antioxidant defenses in the organism with the function of maintaining ROS within physiologically acceptable levels. However, a fraction of the formed ROS recurrently evades this antioxidant control [11]. These oxidant species severely contribute to the skin ageing process, either through direct damage to biomolecules therein, or through interference with signaling pathways within keratinocytes and fibroblasts, thus altering the expression balance of MMPs, procollagen and pro-inflammatory cytokine genes [12,13].

It is known that phenolic compounds, including anthocyanins, which are the major phenolics present in red grapes, not only possess renowned antioxidant properties, but also have the ability to directly inhibit enzymes enrolled in the skin ageing process, namely tyrosinase, collagenase (MMP-1) and elastase [10,14]. In this regard, it is expected that these bioactive compounds will also be encountered in winery residues, such as grape marc, red wine lees and Port wine lees. Grape marc consists of a pressed mixture of grape pulp, skins and seeds, obtained from the separation of the solid fraction of the must from its liquid fraction. Wine lees are the deposit obtained after the fermentation of wine, mainly composed of dead yeast and bacteria, tartaric salts, precipitated tannins, organic and inorganic matter, and free phenolic compounds [15]. The main difference between the vinification process of red wine and Port wine is that, in the latter, alcoholic fermentation is interrupted due to the fortification with wine spirits, which explains the sweetness of some wines and also the high alcoholic grade of Port wine [16].

The work presented herein was focused on the comparative assessment of the cosmeceutical potential of natural extracts obtained from three different winemaking waste streams: Wine lees resulting from alcoholic fermentation of red wine, port wine lees, and red grape marc. For this purpose, all the extracts were characterized in terms of antioxidant activity and inhibitory capacity towards tyrosinase, elastase and MMP-1 (collagenase), through chemical, enzymatic and cell-based assays. Additionally, the phytochemical characterization was carried out by colorimetric assays as well as HPLC-DAD-MS/MS. The main goal of this study was to validate the antioxidant, anti-ageing and skin whitening potential of bioactive compounds recovered from winery byproducts, for their application in the cosmetic industry.

## 2. Materials and Methods

### 2.1. Chemicals

The chemicals used for the determination of the phenolic content and anthocyanin content were gallic acid (Fluka, Steinheim, Germany), sodium carbonate and *Folin-Ciocalteu* reagent (Panreac, Barcelona, Spain), potassium chloride (Sigma-Aldrich, St. Louis, MO, USA), and sodium acetate trihydrate (Sigma-Aldrich, Steinheim, Germany). For HPLC analyses, acetonitrile (Panreac, Barcelona, Spain) and formic acid (VWR-CHEM, Radnor, PA, USA) were used. For the antioxidant activity assays, the reagents were disodium fluorescein, 2,2′-azobis(2-methylpropionamidine)dihydrochloride (AAPH), cobalt (II) fluoride tetrahydrate (Sigma-Aldrich, St. Louis, MO, USA), (+/−)-6-hydroxy-2,5,7,8-tetramethylchroman-2-carboxylic acid (Trolox) (Aldrich, St. Louis, MO, USA), caffeic acid (Sigma-Aldrich, St. Louis, MO, USA), hydrogen peroxide 30 wt. % in water and iron (III) chloride (Sigma-Aldrich, Steinheim, Germany), and acetone (Sigma-Aldrich, St. Louis, MO, USA). For the preparation of phosphate-buffered saline (PBS) 75 mM pH 7.40, potassium phosphate monobasic anhydrous (KH_2_PO_4_) (Amresco, Solon, OH, USA), sodium phosphate dibasic dihydrate (Na_2_HPO_4_•2H_2_O) (Sigma-Aldrich, Steinheim, Germany), potassium chloride (KCl) and sodium chloride (NaCl) (Sigma-Aldrich, St. Louis, MO, USA) were used. Sodium phosphate dibasic dihydrate (Na_2_HPO_4_•2H_2_O) and sodium phosphate monobasic monohydrate (NaH_2_PO_4_•H_2_O) (Sigma-Aldrich, Steinheim, Germany) were used to prepare sodium phosphate buffer solution (SPB) 75 mM pH 7.40. The tyrosinase inhibition was assessed using 3,4-dihydroxy-l-phenylalanine (L-DOPA) (Sigma-Aldrich, St. Louis, MO, USA), mushroom tyrosinase (Sigma-Aldrich, St. Louis, MO, USA), and kojic acid (Sigma-Aldrich, St. Louis, MO, USA). The elastase inhibition was assayed with porcine pancreatic elastase (PPE) type III and N-succinyl-Ala-Ala-Ala-*p*-nitroanilide (AAAPVN) (Sigma-Aldrich, St. Louis, MO, USA); for MMP-1 inhibition reagents were recombinant (expressed in *E. coli*) MMP-1 (Sigma-Aldrich, St. Louis, MO, USA) and MMP fluorogenic substrate (Enzo Life Sciences, Farmingdale, NY, USA). Tyrosinase assay buffer (SPB 0.1 M, pH 6.8) was prepared with sodium phosphate dibasic dihydrate and sodium phosphate monobasic monohydrate (Sigma-Aldrich, Steinheim, Germany); Tris base (Sigma-Aldrich, St. Louis, MO, USA) and hydrochloric acid (HCl) 37% (*w*/*w*) (Honeywell Riedel-de-Häen, Hanover, Germany) were used to prepare elastase assay buffer (Tris-HCl 0.1 M, pH 8); the buffer used in the MMP-1 assay (0.05 M Tris-HCl, pH 7.5) was prepared with Tris-HCl (Fluka, Steinheim, Germany), calcium chloride dihydrate (CaCl_2_•2H_2_O) (Riedel-de Haën, Seelze, Germany), sodium azide (NaN3) (Sigma-Aldrich, Steinheim, Germany), Brij 35 (Fisher Scientific, Geel, Belgium), zinc sulfate heptahydrate (ZnSO_4_•7H_2_O) (Merck, Darmstadt, Germany), and sodium chloride (Sigma-Aldrich, St. Louis, MO, USA). For cell-based assays, the high glucose Dulbecco’s modified eagle medium (DMEM) (Gibco – Thermo Fisher Scientific, Grand Island, NY, USA) and Iscove’s Modified Dulbecco’s Medium (IMDM – GlutaMAX™) (Gibco – Thermo Fisher Scientific, Paisley, UK) were used for cell culturing, and the cells were subcultured with 0.25% trypsin-EDTA (Gibco – Thermo Fisher Scientific, Paisley, UK). Both DMEM and IMDM were supplemented with fetal bovine serum (FBS) (Biowest, Nuaillé, France), and Penicillin-Streptomycin (Gibco – Thermo Fisher Scientific, Grand Island, NY, USA). CellTiter 96^®^ AQueous One Solution Cell Proliferation Assay (MTS/5-(3-carboxymethoxyphenyl)-2-(4,5-dimethylthiazoly)-3-(4-sulfophenyl)tetrazolium, inner salt), from Promega (Madison, WI, USA), was used for cytotoxicity evaluation. For the cellular antioxidant activity assays, 2′,7′-dichlorofluorescin diacetate (DCFH-DA) (Sigma-Aldrich, St. Louis, MO, USA) and tert-butyl hydroperoxide (TBHP) 70% wt. in water (Sigma-Aldrich, Steinheim, Germany) were used.

### 2.2. Samples

The red wine lees from alcoholic fermentation and grape marc of *Tempranillo* grapes from Ribera del Duero Denomination of Origin were kindly provided by Matarromera winery (Valladolid, Spain) in 2015. The port wine lees were generously provided by Sogrape Vinhos S.A. (Porto, Portugal) in 2015.

The extracts from the three different winemaking waste streams were prepared by conventional solid-liquid (SL) extraction, preceded or not by microwave (MW) pretreatment. The grape marc extraction procedure was followed according to a process intensification study described by Álvarez [17], and the optimized extraction parameters for the port and red wine lees were used as designated by Romero-Díez [15]. In brief, the SL extraction of grape marc was carried out with 50:50 (% *v*/*v*) EtOH:H_2_O (water acidified to pH 1 with sulfuric acid) at 60 °C. For MW-pretreated grape marc, the SL extraction was performed at 60 °C with the same extraction solvent after MW irradiation of the samples to achieve a maximum temperature of 80 °C (60 s). The SL extractions from wine lees were performed at 25 °C with 50:50 (% *v*/*v*) EtOH:H_2_O (water acidified to pH 2.5 with hydrochloric acid). For MW-pretreated wine lees, the sample (with a solvent mixture of 60:40 (% *v*/*v*) EtOH:H_2_O) was irradiated for 90 s to reach a temperature of 115 °C; immediately after, the mixture was cooled down and additional solvent was added to carry out the extraction at 25 °C as described above. All chosen extraction temperatures resulted from an optimization process aiming at maximizing the extraction of anthocyanins from grape marc and wine lees, which are the main phenolics present in these raw materials. These optimization studies [15,17,18] took into consideration the compromise between the thermal effect that enhances the extraction yield of phenolic compounds while reducing the extraction time, and the vulnerability of phenolics to thermal degradation. In the case of MW-pretreatment, it was concluded that the high temperatures achieved after irradiation did not lead to degradation of phenolics due to the short duration of the heating. The MW pretreatments were carried out in a CEM Discover Microwave (CEM Corporation, Matthews, NC, USA) using a maximum power of 300 W. Ethanol was eliminated from the samples by rotary vacuum evaporation until only 5% ethanol was achieved, and phytochemical as well as antioxidant activity characterization was performed. For the enzymatic and cell-based assays, the extracts were dried in a CentriVap Concentrator (Labconco, Kansas City, MO, USA), solubilized in DMSO and then stored at −20 °C.

### 2.3. Methods

#### 2.3.1. Phytochemical Characterization

##### Total Phenolic Content (TPC)

The total phenolic content of the extracts was determined by the Folin-Ciocalteu (FC) method, relying on the electron transfer from the phenolic compounds to phosphomolybdic/phosphotungstic acid complexes in alkaline medium. This resulted in the formation of blue complexes which absorbance at 765 nm is proportional to the amount of phenolics [19]. This assay was based on previous work [20], by adding FC reagent and a saturated sodium carbonate solution to the samples, and adapted for a Spark 10M (Tecan Group Ltd., Zürich, Switzerland) spectrophotometer microplate reader. The absorbances were measured against the blank and the TPC values were calculated from a gallic acid standard curve and expressed as milligrams of gallic acid equivalents (GAE) per gram of dry extract.

##### Total Anthocyanin Content (TAC)

The total monomeric anthocyanin pigment content was assessed by a pH differential method, following the protocol described in the AOAC Official Method 2005.02 [21]. This method relies on the color change of monomeric anthocyanin pigments depending on the pH, and their differential absorbance at 520 nm. The readings were carried out in a Spark 10M (Tecan Group Ltd., Zürich, Switzerland) spectrophotometer microplate reader, and the results were calculated as milligrams of malvidin-3-O-glucoside equivalents per gram of dry extract, since this anthocyanin is a major compound in all the extracts.

##### High Performance Liquid Chromatography–Mass Spectrometry (HPLC-DAD-MS/MS)

The samples were analyzed by HPLC-DAD-MS/MS, using a Waters Alliance 2695 Separation Module (Waters, Ireland) system equipped with a quaternary pump, a degasser, an autosampler and a column oven. The liquid chromatography system was coupled to a photodiode array detector 996 PDA, and to a mass spectrometer MicroMass Quattromicro^®^ API (Waters, Ireland). All data were acquired and processed by MassLynx^®^ 4.1 software.

The chromatographic separation of compounds was carried out in a reversed-phase LiChrospher^®^ 100 RP-18 5µm LiChroCART^®^ 250-4 column inside a thermostatic oven at 35 °C. A binary mobile phase was used, at a flowrate of 0.3 mL/min, with eluent A composed of formic acid (0.5% *v*/*v* in ultrapure water) and eluent B of acetonitrile. The gradient program used was 99:1 A:B for 5 min, from 99:1 A:B to 40:60 A:B in 40 min, from 40:60 A:B to 10:90 A:B in 45 min, held isocratically (90% B) for 10 min, from 10:90 A:B to 99:1 A:B in 10 min, and finally held isocratically (99:1 A:B) for 10 min. An injection volume of 20 µL was used. The absorption spectra were acquired from 210 to 600 nm by a photodiode array detector. Mass spectrometry was performed using an electrospray ion source in the negative and positive ion mode, with the temperature set at 120 °C, the capillary voltage at 2.5 kV, and the source voltage at 30 V. The compounds separated by HPLC were ionized and the mass spectra were recorded in a full scan mode, with m/z range between 100 and 1500. High purity nitrogen was used as drying and nebulizing gas, and ultrahigh purity argon was used as collision gas.

#### 2.3.2. Antioxidant Activity

The antioxidant activity of the extracts was assessed towards different ROS, through three complementary antioxidant assays: Oxygen radical absorbance capacity (ORAC), hydroxyl radical scavenging capacity (HOSC), and hydroxyl radical averting capacity (HORAC). All three assays rely on the capacity of the samples to prevent the oxidation of disodium fluorescein (FL). In all cases, fluorescence (Ex/Em 485 ± 20/528 ± 20 nm) emitted by the reduced form of FL was recorded over time, at 37 °C, in a FL800 microplate fluorescence reader (Bio-Tek Instruments, Winooski, VT, USA), under the control of Gen5 software.

The ORAC evaluates the antioxidant capacity of the tested samples towards peroxyl radicals (ROO^•^) generated during thermal decomposition of AAPH. This assay was based in the method developed by Huang [22], with some modifications. Briefly, FL was added to sample dilutions and the resulting mixture was equilibrated to 37 °C, then, the reaction was initiated by the addition of AAPH and fluorescence was recorded for 40 min. Final concentrations in the reaction mixture were 2.25 × 10^−4^ mM FL and 19.12 mM AAPH, and all solutions were prepared in a phosphate-buffered saline (PBS), 75 mM, pH 7.4.

The HOSC estimates the capacity of the samples to scavenge hydroxyl radicals (^•^OH) generated from a Fe(III)-driven Fenton-like reaction. The assay was performed as described by Moore [23], with slight modifications. Briefly, FL, hydrogen peroxide (H_2_O_2_) and iron (III) chloride (FeCl_3_) were added to sample dilutions, and fluorescence was measured for 60 min. The final concentrations of the reagents were 5.64 × 10^−5^ mM FL, 26.67 mM H_2_O_2_ and 0.68 mM FeCl_3_. FL solution was prepared in a sodium phosphate buffer (SPB), 75 mM, pH 7.4, FeCl_3_ and H_2_O_2_ solutions were prepared in MilliQ water, while sample dilutions were made in Acetone:MilliQ water 50% (*v*/*v*).

The HORAC aims to evaluate the capacity of a given sample to prevent the generation of hydroxyl radicals (^•^OH) by a Co(II)-mediated Fenton-like reaction. The procedure was performed based on the method described by Ou [24], with some modifications. In brief, FL, hydrogen peroxide (H_2_O_2_) and cobalt (II) fluoride (CoF_2_) were added to the sample dilutions, and fluorescence was measured for 60 min. The final concentrations were 5.64 × 10^−5^ mM FL, 26.67 mM H_2_O_2_ and 0.41 mM CoF_2_. The FL solution was prepared in a sodium phosphate buffer (SPB), 75 mM, pH 7.4, CoF_2_ and H_2_O_2_ solutions were prepared in MilliQ water, while the sample dilutions were made in Acetone:MilliQ water 50% (*v*/*v*).

The ORAC and HOSC values were calculated from a trolox standard curve and expressed as micromoles of Trolox equivalents (TE) per gram of dry extract, whereas for HORAC, caffeic acid was used as the standard and the results were expressed as micromoles of caffeic acid equivalents (CAE) per gram of dry extract. In all three assays, the calculations took into consideration the dilution effect on the antioxidant capacity [25].

#### 2.3.3. Enzymatic Assays

##### Inhibition of Tyrosinase

The tyrosinase inhibitory capacity of the extracts was determined spectrophotometrically, using mushroom tyrosinase and L-DOPA as the substrate, as reported in the literature [26]. Tyrosinase converts L-DOPA to Dopaquinone, which in turn cyclizes to form Dopachrome. The dopachrome formation can be monitored by measuring the absorbance at 475 nm. Shortly after, L-DOPA was added to tyrosinase in the presence of the sample dilutions, to a final concentration of 6 U/mL tyrosinase and 0.5 mM L-DOPA. After a 30 min incubation at 37 °C, absorbance was measured at 475 nm in a Spark 10M (Tecan Group Ltd., Männedorf, Zürich, Switzerland). All reagents were prepared in SPB, 0.1 M, pH 6.8. The calculations were made as follows:

(1) $$\%\;inhibition=\frac{\left(Acontrol-Asample\right)}{Acontrol}*100$$

where A_control_ and A_sample_ stand for the A_475_ in the absence or presence of the sample, respectively. The inhibitory potential of the extracts was evaluated with increasing concentrations, in order to establish dose-dependent relationships and determine the half maximal inhibitory concentration (IC_50_) values, meaning the capacity of the samples to inhibit the enzymatic activity to an extent of 50%.

##### Inhibition of Elastase

The elastase inhibitory capacity of the extracts was determined by a colorimetric assay, using porcine pancreatic elastase (PPE) and N-succinyl-Ala-Ala-Ala-p-nitroanilide (AAAPVN) as the substrate. Notably, *p*-nitroaniline is formed after cleavage of the substrate and its formation can be monitored by measuring the absorbance at 410 nm. Briefly, elastase was added to the sample dilutions, and after equilibrating the temperature to 25 °C for 20 min, the reaction was initiated by the addition of the substrate. After a 20 min incubation, absorbance was measured at 410 nm in a Spark 10M (Tecan Group Ltd., Männedorf, Zürich, Switzerland). The procedure was carried out in a Tris-HCl buffer (0.1 M, pH 8), and the final concentrations were 0.03 U/mL elastase and 0.05 mg/mL AAAPVN. The calculations were made as described in Equation (1), and several concentrations of the extracts were tested in order to determine the IC_50_ values.

##### Inhibition of MMP-1

The capacity of the extracts to inhibit matrix metalloproteinase-1 (MMP-1) was assayed using human recombinant MMP-1, and a fluorogenic peptide as MMP substrate, displaying strong fluorescence (Ex/Em 340/440 nm) once cleaved by the enzyme. Briefly, the fluorogenic substrate was added to MMP-1 in the presence of the extract dilutions, with the final concentrations of 0.2 µg/mL MMP-1 and 1 µM fluorogenic substrate. The reaction was allowed to occur for 20h at 37 °C, and then fluorescence was measured in a multimode microplate reader (Spark 10M, Tecan Group Ltd., Männedorf, Zürich, Switzerland). Tris-HCl 50 mM, pH 7.5, with 10 mM CaCl_2_, 150 mM NaCl, 0.02% (*w*/*v*) NaN_3_, 0.05% (*w*/*v*) Brij 35 and 0.05 mM ZnSO_4_ was used as the assay buffer, according to the literature [27]. The calculations were made as described in Equation (1), and several concentrations of the extracts were tested in order to determine the IC_50_ values.

#### 2.3.4. Cell-based Assays

##### Cell Culture

The human immortalized non-tumorigenic keratinocyte cell line HaCaT (CLS, Germany) was cultured in high glucose, high pyruvate, Dulbecco’s modified eagle medium (DMEM), whereas the human foreskin fibroblasts (HFF) cell line CCD-1112Sk (ATCC, USA) was cultured with Glutamax™ Iscove’s Modified Dulbecco’s Medium (IMDM). Both culture media were supplemented with 10% (*v*/*v*) heat-inactivated fetal bovine serum (FBS), 100 units/mL penicillin and 100 µg/mL streptomycin. All experiments were performed in culture media supplemented with only 0.5% FBS and no antibiotic. For every assay, the cells were seeded in 96-well TC (tissue culture)-treated microplates at a density of 1.4 × 10^5^ cells/cm^2^ (HaCaT) or 3.1 × 10^4^ cells/cm^2^ (HFF) and allowed to reach confluence. The cells were cultured in a humidified atmosphere at 37 °C with 5% CO_2_.

##### Cytotoxicity Evaluation

In order to determine the nontoxic concentrations of the extracts for further studies, the cells were exposed to several concentrations of the extracts diluted in culture medium for 24 h. The well content was then removed, and the cells were washed twice with PBS. A solution of 1.6% *v*/*v* MTS in the medium was added to the cells for 3 h, and absorbance was measured at 490 nm in a multimode microplate reader (Spark 10M, Tecan Group Ltd., Männedorf, Zürich, Switzerland). Cell viability was determined as a percentage of control, after blank subtraction. The MTS assay is based on the reduction of a tetrazolium salt by viable cells to generate a colored, aqueous soluble formazan product, of which absorbance can be measured at 490 nm. The amount of formazan produced is directly proportional to the number of viable cells.

##### Cellular Antioxidant Activity

The capacity of the extracts to inhibit ROS production in the cells was evaluated using two different approaches: Pre-incubation and co-incubation. In both cases, 2′,7′-dichlorofluorescin diacetate (DCFH-DA) was used as a fluorescent probe. Non-fluorescent DCFH-DA readily diffuses through the cell membrane and once in the intracellular medium, the diacetate moiety is cleaved by cellular esterases giving rise to the more polar 2′,7′-dichlorodidhydrofluorescein (DCFH_2_) which remains trapped within the cell. ROS from intrinsic oxidative stress or generated by an oxidative stress inducer easily diffuse into the cell, where they oxidize DCFH_2_ to its fluorescent form, 2′,7′-dichlorofluorescein (DCF). The accumulation of DCF in the cells may be measured by an increase in fluorescence (Ex/Em 485/528 nm), which is proportional to the amount of ROS [28].

In the pre-incubation approach, cells were treated with selected non-toxic concentrations of the samples for 1 h or 24 h (cytotoxicity data in Supplementary Material, Figures S1 and S2), and then incubated with 25 µM DCFH-DA in PBS for 1 h. Fluorescence was measured at this point in order to assess the antioxidant effect of the samples towards intrinsic ROS. DCFH-DA was then removed and a non-cytotoxic concentration of *tert*-butyl hydroperoxide (TBHP) was added to the cells in PBS (0.625 mM for HaCaT and 1.25 mM for HFF). After 1 h, fluorescence was measured. In the co-incubation approach, the cells were incubated with 25 µM DCFH-DA for 1 h in PBS, and then the chosen concentrations of the stress inducer and extract were simultaneously added to the cells, in PBS. After 1 h, fluorescence was measured. All the results were presented as fluorescence percentages relative to the untreated control. Fluorescence measurements were performed in a FL800 microplate fluorescence reader (Bio-Tek Instruments, Winooski, VT, USA), with fluorescence filters (Ex/Em 485 ± 20/528 ± 20 nm).

##### Protection against Oxidant-Induced Cytotoxicity

To assess the potential of the samples to prevent TBHP-induced cytotoxicity, the cells were incubated with selected non-toxic concentrations of the extracts for 24 h. After this period, the cells were incubated for 1 h with a concentration of TBHP capable of inducing cytotoxicity (20 mM for HaCaT and 10 mM for HFF), and then cell viability was measured using the MTS assay. The absorbance measurements were performed in an EPOCH 2 microplate reader (Bio-Tek Instruments, Winooski, VT, USA).

#### 2.3.5. Statistical Analysis

All the results are expressed as the mean ± standard deviation (SD), obtained from at least three independent experiments. Statistical analysis of the results was performed using GraphPad Prism 6 software (GraphPad Software, Inc., La Jolla, CA). When homogeneous variance was confirmed, the results were analyzed by one-way analysis of variance (ANOVA), followed by the Tukey test for multiple comparisons. In the case of heterogeneous variances, an appropriate unpaired student *t*-test was performed in order to determine whether the means were significantly different. A *p*-value ≤ 0.05 was accepted as statistically significant in all cases.

## 3. Results and Discussion

### 3.1. Phytochemical and Antioxidant Activity Characterization

#### 3.1.1. Total Phenolic Content (TPC) and Total Anthocyanin Content (TAC)

In a first approach, this study aimed to assess the effect of MW-pretreatment in the extraction methodology, by characterizing the extracts in terms of total phenolic content (TPC). Moreover, since the extracts studied in this work were obtained from red grape marc and red wine lees, and given anthocyanins are a relevant class of compounds encountered in these matrices, total anthocyanin content (TAC) was also evaluated.

Microwaves (MW) have been widely used to assist the extraction of several compounds from plant matrices, as they generally result in higher extraction yields, shorter extraction times, and reduced amounts of solvent needed, when compared to conventional extraction techniques. This is because water molecules present in the matrix absorb MW energy, leading to rapid heating and evaporation of intracellular water, which in turn causes disruption of the plant cell membrane-limited compartments, improving the mass transfer process of substances of interest from the raw material to the extraction solvent [29,30]. To overcome the scale-up limitations of a full-time low frequency microwave-assisted extraction (MAE), particularly the non-uniform irradiation of a large vessel, MW-pretreatment has been suggested, which comprises a short time irradiation with a higher MW frequency that allows the material to be homogeneously irradiated. The short duration (<120 s) peak of energy obtained in the MW-pretreatment is proposed to avoid degradation of the active compounds while maintaining the MW thermal effect that accelerates the extraction [17].

In both the Port and red wine lees extracts, the higher values of TPC and TAC are observed in the case of MW-pretreated matrices (Table 1), implying that MW treatment prior to SL extraction positively influences (poly)phenol and anthocyanin richness. This finding agrees with the abovementioned principle of MW-pretreatment, and equivalent results have been reported in the literature for the same matrices [15]. It was found that TAC in MW80 GM (2.7 mg malv-3-O-gl/g extract) was significantly higher (>1.5-fold increase) than GM (1.7 mg malv-3-O-gl/g extract). On the other hand, the grape marc conventional extract (GM) presents a higher TPC (83.9 mg GAE/g extract) than the grape marc extract obtained after MW-pretreatment (45.9 mg GAE/g extract). These results suggest that the energy of the MW-pretreatment used may have not been enough to improve the extraction of all subclasses of phenolics, but instead selectively increased anthocyanin richness. When comparing our results with the ones obtained by Álvarez [17], it is evident that the order of magnitude for both TPC and TAC of grape marc extracts studied herein is much lower. This may be explained by the usage of different batches of grape marc (2014 versus 2015 vintage) and thus the different composition of the raw material used, since crops from different years may present considerable variability in sugar content and nutritional composition. Nevertheless, the trend observed for TAC agrees with the one found for grape marc in the literature [17].

Still, the red wine lees extracts, both with and without MW pretreatment (MW RW and RW, respectively), are the richest of the studied extracts in terms of both TPC and TAC. In particular, the TPC values of the red wine lees extracts are at least two-fold higher than those of the Port wine lees extracts, and three-fold higher than those of the grape marc extracts, whereas the TAC values of the red wine lees extracts are at least two-fold higher than those of the Port wine lees extracts and ten-fold higher than those of the grape marc extracts. These results may be due to the sugars found in the Port wine and grape marc, that are extracted along with phenolics, leading to a reduced (poly)phenol richness per mass of extract when compared to the red wine lees extracts. Additionally, the grape marc extracts have significantly lower TAC values than both wine lees extracts, probably because of the higher availability of anthocyanins in wine lees due to the extraction of phenolics from grape skin taking place in the winemaking process [31]. Moreover, it is known that the capacity of wine lees to adsorb colorants leads to a concentration of anthocyanins up to ten times higher than in red grape skin [32].

#### 3.1.2. Identification of Compounds by HPLC-MS/MS Analysis

An HPLC apparatus coupled to a diode array detector (DAD) and a mass spectrometer (MS) was used to identify the main compounds present in the three raw materials used. The identification was carried out taking into consideration the absorption of compounds at four wavelengths (280, 320, 360 and 520 nm, for phenolics in general, phenolic acids, flavonols, and anthocyanins, respectively), the *m*/*z* peaks corresponding to precursor and daughter ions, a comparison of chromatographic profiles with those of standard compounds, databanks [33,34], and studies already reported in the literature for comparable matrices [15,35,36,37,38,39,40].

The TPC and TAC obtained by this method were consistent with the results obtained by the colorimetric assays, in terms of relative quantification. The relative flavonol amounts in the extracts were also estimated by calculating the total peak area of the chromatograms at 360 nm, and it was found that the order of flavonol content was: RW > MW RW > MW P > P > MW80 GM > GM.

**Figure 1:**
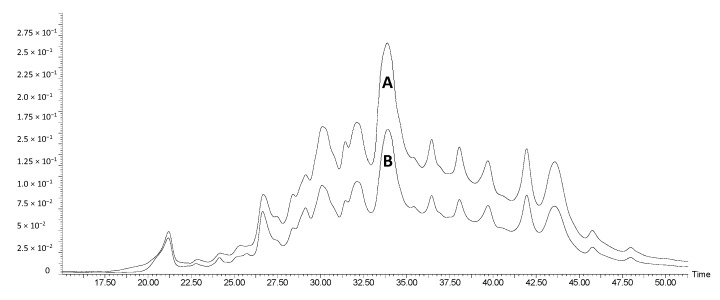
Chromatograms at 280 nm of MW RW (**A**) and RW (**B**), as obtained by HPLC.

In all cases, the extracts obtained from the same matrix had the same qualitative composition, differing only in quantitative composition, depending on the extraction methodology. An example is presented in Figure 1 for RW and MW RW, corroborating the positive effect of MW-pretreatment on the amount of phenolics extracted. Since the chromatographic profiles of the extracts obtained from the same waste stream matrices are qualitatively identical, the identifications presented in Table 2 are organized by raw materials, along with m/z values, fragment ions and phenolic subclasses. The qualitative composition of the different raw materials is similar, however, some differences can be pointed out. In particular, some anthocyanin conjugates (petunidin-, malvidin-, and peonidin-3-O-6”-*p*-acetylglucosides), that were identified in the Port and red wine lees, were not detected in grape marc. Once again, this may be explained by the higher availability of phenolics in wine lees rather than in grape marc. However, two pyranoanthocyanins (vitisin A and 10-carboxypyranomalvidin-3-6”-*p*-coumaroylglucoside), resulting from the reactions between malvidin-3-O-glucoside and pyruvic acid, were identified in the three raw materials. These anthocyanin-derived compounds can only be formed after alcoholic fermentation occurs, as pyruvic acid is a product of this reaction [38]. Since grape marc is only separated after the alcoholic fermentation is initiated, it is plausible that these pigments are found, not only in wine lees, but also in grape marc. These findings are corroborated by literature, since vitisin A has already been identified in Port and red wine lees [15], and several pyranoanthocyanins, including vitisin A and 10-carboxypyranomalvidin-3-6”-*p*-coumaroylglucoside, have been detected in red grape marc [41].

#### 3.1.3. Antioxidant Activity Characterization

Phenolics are well known for their antioxidant activity, which is an important feature that determines the relevance of these compounds for cosmetic applications, given that ROS are the driving causes of skin ageing. Therefore, the extracts under study were submitted to three complementary antioxidant assays, aiming at assessing the antioxidant capacities of the samples towards different biologically relevant radical species, namely hydroxyl (^•^OH) and peroxyl (ROO^•^) radicals. Hydroxyl radicals are primarily generated from Fenton-like reactions, in which metal ions are oxidized by hydrogen peroxide (Metal^2+^ + H_2_O_2_→Metal^3+^ + ^•^OH + OH^−^), and are highly reactive species that are able to oxidize numerous biomolecules, including membrane lipids by initiating lipid peroxidation through the abstraction of hydrogen atoms from unsaturated fatty acids, resulting in the generation of peroxyl radicals (ROO^•^) [11].

The antioxidant activity results summed up in Table 1 show that in all extracts, there are compounds exerting the three types of antioxidant activity: Peroxyl and hydroxyl radicals scavenging, and transition metal ion chelation. The red wine lees extracts (RW and MW RW) presented significantly better results than all the other extracts in all three assays, with antioxidant capacity >3000 µmol TE/g extract in ORAC and HOSC, and HORAC values >1900 µmol CAE/g extract (Table 1). In the case of the Port wine lees extracts, MW P had significantly higher potential for peroxyl and hydroxyl radical scavenging as well as metal ion chelation, presenting higher ORAC, HOSC and HORAC values than P. Among the grape marc extracts, MW80 GM revealed lower antioxidant capacity than GM in HOSC and HORAC but not in ORAC.

The ORAC, HOSC and HORAC values correlated well with TPC, TAC and the relative amounts of flavonols for all samples, with R^2^ values ≥0.95. Although the higher amounts of phenolics correspond to a more promising antioxidant activity, the different proportions of phenolic subclasses in the extracts may also lead to different activities towards distinct oxidant sources. For instance, flavonoids are generally more capable of inactivating peroxyl radicals than small phenolic antioxidants, whereas monohydroxybenzoic acids are very effective in the inactivation of hydroxyl radicals [42]. However, in this work, the antioxidant activity results seem to correlate well (R^2^ > 0.8) with most of the compounds identified in the studied samples.

Similar to what has been observed for TPC, the ORAC values for the grape marc extracts were much lower than the ones described by Álvarez [17] (a decrease higher than 60% was observed for the two extracts), which may be justified, once again, by different vintages (2014 versus 2015). Nonetheless, the MW pretreated grape marc yielded the same ORAC values as the respective conventional extract, which is in agreement with Álvarez [17]. Regarding Port and red wine lees, the conventional extracts presented the same ORAC values as those reported in previous work [15]. Although for red wine lees, the extract obtained following MW pretreatment did not reveal a significantly higher ORAC value than the corresponding conventional extract, in the case of Port wine lees, the MW-pretreatment increased ORAC. Regarding HOSC and HORAC, similar results as the ones obtained for red wine lees were found in the literature for a similar matrix (ageing wine lees) [40].

The antioxidant activity values (as well as TPC and TAC) found in the literature for grape marc and wine lees of different origin as the ones used in this work are highly variable, and a comparison with the ones obtained herein is not easy because these determinations greatly depend on the grape variety, maturation stage, environmental conditions during grape growth, vinification parameters, and extraction procedure.

### 3.2. Screening of the Cosmetic Potential of Wine Lees and Grape Marc Extracts

#### 3.2.1. Anti-Hyperpigmentation Activity

##### Inhibition of Tyrosinase

Along with ageing, pigmentation disorders tend to appear in the skin, which has attracted the attention of cosmetic industries and led the quest to find compounds with anti-hyperpigmentation potential. These pigmentation lesions are caused by alterations resulting in the accumulation of melanin, thus the inhibition of melanin production is the most explored approach in this field. As tyrosinase is the rate-limiting enzyme in melanin synthesis, it is a promising target for the development of skin-whitening cosmetic products. Due to their aromatic structural features, phenolics bear some similarities to tyrosine, the substrate of tyrosinase that initiates the synthesis of melanin. Hence, phenolics are potential analogs of tyrosine that can act as competitive inhibitors. In addition, tyrosinase contains a copper ion in its active site, and certain phenolics have the ability to chelate transition metal ions [43].

All tested extracts showed a dose-dependent inhibiting effect on tyrosinase, which allowed for the determination of the IC_50_ values (Table 3). The wine lees extracts presented lower IC_50_ values (≤1.06 mg extract/mL) than the grape marc extracts (≥4 mg extract/mL), which means that the former are more potent inhibitors of tyrosinase than the latter. Amongst the wine lees matrices, red wine lees (RW and MW RW) showed the best results, with the highest potential for tyrosinase inhibition (IC_50_ ≤ 0.2 mg extract/mL). The capacity of the extracts to inhibit tyrosinase does not correlate directly with TPC, TAC or the relative amount of flavonols (R^2^ ≤ 0.64), suggesting that the capacity to inhibit tyrosinase relies more on the presence of specific compounds rather than the overall amount of phenolics. For instance, GM exhibited a TPC almost two-fold higher than MW80 GM (83.9 versus 45.9 mg GAE/g extract), yet these two extracts revealed the same IC_50_ towards tyrosinase.

The inhibition of tyrosinase may take place by the direct interference of phenolics with the active site, the allosteric interactions leading to conformational changes or the loss of function, or by copper ion chelation. Flavonoids possess metal-binding motifs in their structure [43], hence these phenolics may play a significant role in tyrosinase inhibition. The areas of the peaks obtained from the mass spectra, corresponding to the compounds identified in the samples, allowed for the calculation of correlation coefficients between the amount of each compound and the effectiveness of the extract in inhibiting tyrosinase. It was found that, although the correlations were not very high with any of the identified compounds, tyrosinase inhibition correlated better with syringetin-3-O-glucoside (R^2^ = 0.69), myricetin (R^2^ = 0.66) and malvidin-3-O-glucoside (R^2^ = 0.65) than with all the other identified compounds (R^2^ ≤ 0.48). In the literature, myricetin has already been described as a tyrosinase inhibitor [44]. Nevertheless, other compounds present in the extracts, in particular other flavonoid aglycones, may be contributing as well for the exhibited tyrosinase inhibitory activity, since quercetin, kaempferol, and catechins, among other, are also reported to be effective tyrosinase inhibitors [44].

Kojic acid is a well-studied inhibitor of tyrosinase that is widely used as a reference for a comparison with novel potential inhibitors of the enzyme [45]. Kojic acid’s IC_50_ was 0.03 mg/mL, and the red wine lees extracts effectiveness came remarkably close, particularly MW RW, presenting an IC_50_ value only of approximately five-fold higher than kojic acid, which is more promising than several other plant extracts [46].

#### 3.2.2. Anti-ageing Activity

##### Inhibition of Elastase

Elastases contribute to the reduced amount of elastin in the skin by degrading not only the elastic fibers therein, but also newly formed elastin, hampering its correct assembly into functional elastic fibers. Moreover, elastases are broadly specific enzymes being also able to degrade other ECM proteins. Therefore, studying the capacity of the extracts to inhibit elastase is relevant for validating their anti-ageing potential. The IC_50_ values are displayed in Table 3 and, following the trend of previous assays, the red wine lees extracts presented the lowest IC_50_ values (≤0.17 mg extract/mL). The correlations of elastase IC_50_ values with TAC and the relative amounts of flavonols were not very high (R^2^ ≤ 0.53) and, although the relationship between IC_50_ and TPC is not linear (R^2^ = 0.66), there is a direct correspondence between these two parameters, which suggests that the amount of phenolics determines the inhibitory capacity of the extracts. In fact, it has been reported that higher TPC leads to stronger inhibition of elastase [10]. The inhibition of elastase relies on van der Waals (vdW) interactions and hydrogen bonds between the enzyme and the inhibitor, thus phenolics with a larger number of potential interaction sites, including aromatic rings for vdW interactions and hydroxyl groups for hydrogen bonding, are more likely to better inhibit elastase. The structural features playing an important part in the inhibitory capacity of phenolics towards elastase are the galloyl moiety, the degree of polymerization in the case of procyanidins, and hydroxylation of the structure [47,48]. However, the presence of glycosidic groups in the phenolic structure was found to preclude the inhibitory interaction with the enzyme, due to steric hindrance, suggesting that flavonoid aglycones may be more relevant for the elastase inhibitory capacity of the extracts than their respective glycoside derivatives [10,48]. Wittenauer [10] reported that gallic acid, catechin and procyanidins exhibited inhibitory activity against elastase, and Sartor [48] studied the effect of several compounds for this purpose, including myricetin and quercetin. Therefore, the authors suggest that gallic acid, catechin, epicatechin, procyanidin dimers and trimer, as well as the flavonol aglycones identified in the extracts (myricetin, quercetin, kaempferol and rhamnetin) are important in the capacity of winemaking waste streams extracts to inhibit elastase activity. Nonetheless, the IC_50_ values did not correlate particularly well with any specific compound (R^2^ ≤ 0.53), reinforcing the fact that the overall TPC is the most relevant feature for elastase inhibition.

##### Inhibition of MMP-1

Matrix metalloproteinase-1 (MMP-1), also known as interstitial collagenase, is one of the most important enzymes participating in the process of skin ageing. MMP-1 can initiate the degradation of fibrillar types of collagen, such as collagens I and III which are the predominant forms existing in skin, paving the way for further degradation by other enzymes. MMP-1 can also cleave non-fibrillar collagen and other ECM constituents. MMPs have a conserved methionine and a zinc-binding motif in their active site, as well as a similar fold [49], thus the inhibitory capacity of the extracts against MMP-1 may provide an insight into their capacity to inhibit other MMPs. The inhibition of collagenase can be achieved by either unspecific non-covalent interactions with amino acid side chains leading to conformational changes, interaction with the binding site, or complexation of the zinc ion (Zn^2+^) present in the catalytic site [10]. The most effective inhibitor would combine both metal ion chelation and interactions with the protein through vdW forces and hydrogen bonds.

The IC_50_ values of MMP-1 inhibition by the extracts are displayed in Table 3. Once again, red wine lees presented the best results in terms of inhibitory capacity towards MMP-1 (IC_50_ ≤ 0.22 mg extract/mL). A considerably linear relationship (R^2^ = 0.93) between TPC and the IC_50_ values for MMP-1 is observed, which makes sense considering the lack of specificity involved in collagenase inhibition. Additionally, the correlations with TAC and with the relative amounts of flavonols were also good (R^2^ = 0.92 and 0.89, respectively). In fact, several compounds from different phenolic subclasses have been shown to inhibit collagenase and/or other MMPs. Among them, catechins and procyanidins, gallic acid, delphinidin aglycone, myricetin, quercetin, and kaempferol. Further, the important structural features contributing to the inhibition of MMPs include the presence of galloyl moieties, polyhydroxylation of the flavonoid backbone, planarity of the molecule, and the presence of metal-binding motifs have been described [48]. Interestingly, the IC_50_ values for MMP-1 showed particularly good correlations with the compounds from different phenolic subclasses, namely caftaric acid (R^2^ = 0.89) belonging to the class of phenolic acids, the anthocyanins malvidin- (R^2^ = 0.92) and peonidin-3-O-glucoside (R^2^ = 0.89), and the flavonols, myricetin (R^2^ = 0.88) and quercetin (R^2^ = 0.84). Therefore, these might be the main compounds responsible for the MMP-1 inhibitory capacity of the tested extracts, by being able to not only establish vdW interactions and hydrogen bonds with the protein, but also to chelate transition metal ions, including Zn^2+^.

#### 3.2.3. Cellular Antioxidant Activity

The chemical antioxidant assays are useful tools for an initial antioxidant activity screening of compounds or natural extracts. However, these methods have some limitations concerning the prediction of the antioxidant activity of tested samples in a biological environment. The parameters like bioavailability, cellular uptake, and metabolism are not taken into consideration in chemical assays given the simplicity of these systems. For instance, two compounds may have similar antioxidant activities as determined by chemical assays, yet one may be more promising than the other when applied in a biological context because of its availability at the site of action. The cellular antioxidant assays comprise of some of the complexity of biological systems, namely cellular uptake, subcellular location, and metabolism [28]. Since keratinocytes and fibroblasts are the predominant cell types encountered in the skin, representing the epidermal and dermal layers, respectively, the cellular assays were based on keratinocyte (HaCaT) and fibroblast (HFF, CCD-1112Sk) cell lines. These cell types are responsible for skin integrity and, when affected by senescence or oxidative stress, are leading players in the emergence of the aged skin phenotype.

Only the most promising extracts from each raw material were chosen to proceed to cell-based assays, and the selection was based on the results obtained in chemical and enzymatic assays. These results are summarized in Table 4, in which the extracts are rated according to their performance in each assay. MW P and MW RW were chosen due to the overall better results than the respective conventional extracts in the previous experiments. Concerning the grape marc extracts, GM was generally more promising than MW80 GM. However, MW80 GM showed a higher TAC than GM (Table 1), thus both GM and MW80 GM were selected for cell-based assays.

**Figure 2:**
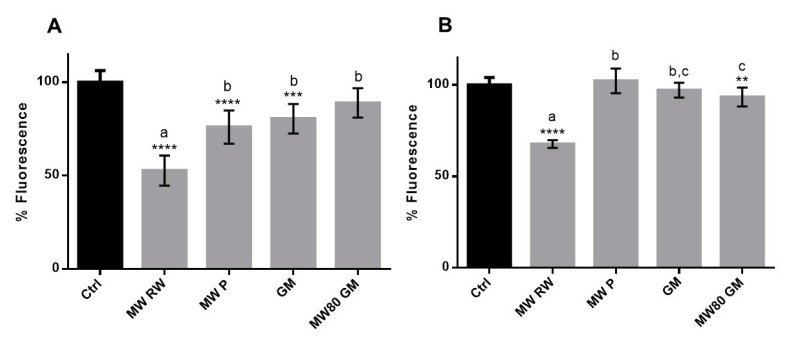
Pre-incubation of cells with 0.25 mg/mL of extracts for 1 h – effect on endogenous ROS. (**A**) HaCaT; (**B**) HFF. The symbol * indicates significance relative to the control (* *p*-value ≤ 0.05, ** *p*-value ≤ 0.01, *** *p*-value ≤ 0.001, **** *p*-value ≤ 0.0001). Statistical differences (*p* ≤ 0.05) between the samples are identified with different letters. MW RW—MW-pretreated red wine lees extract; MW P—MW-pretreated Port wine lees extract; GM—grape marc conventional extract; MW80 GM—MW-pretreated grape marc extract (max. temp. 80 °C).

Primarily, the potential cytotoxicity of the extracts was evaluated in both HaCaT and HFF (Figures S1 and S2) in order to select the non-toxic concentrations for further studies. Then, as a first approach, the capacity of the extracts to inhibit endogenous ROS was evaluated after an incubation period of 1 h. Several concentrations of each extract were tested, and a dose-dependent relationship was observed for all the extracts in both cell lines and incubation periods (Figure S3). However, for comparison purposes, only the common concentration amongst all extracts (0.25 mg extract/mL) is shown in Figure 2. The differences between the same concentrations of different extracts may be justified by their TPC as well as their diverse composition, presenting distinct amounts of phenolics with the ability to permeate or interact with cell membranes. In fact, the correlations between the inhibition of endogenous ROS production and TPC, TAC and the relative amount of flavonols were fairly good (R^2^ ≥ 0.91 in HaCaT, and R^2^ ≥ 0.71 in HFF). Nevertheless, MW RW was the most promising extract, resulting in protection percentages of ≥32%, as opposed to ≤24% observed for other extracts, in both cell lines.

**Figure 3:**
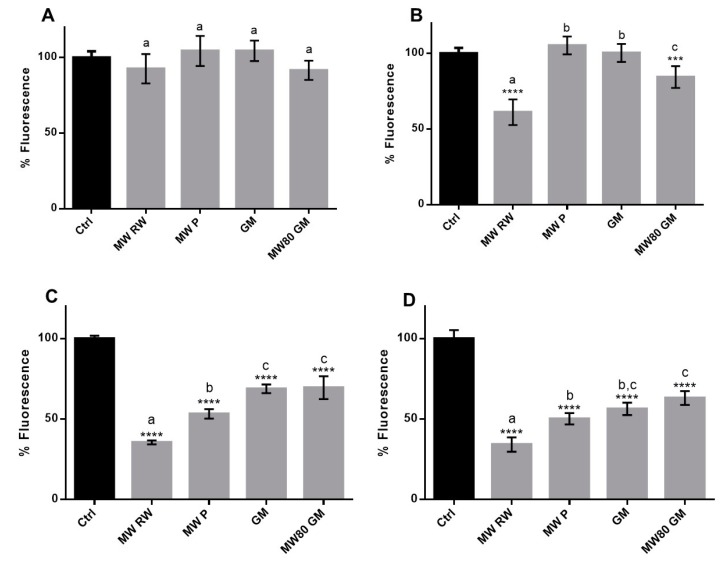
Pre-incubation of cells with 0.25 mg/mL of extracts for 1 h prior to addition of TBHP – effect on induced ROS – in HaCaT (**A**) and HFF (**B**); co-incubation of HaCaT (**C**) and HFF (**D**) with extracts and TBHP for 1 h. The symbol * indicates significance relative to the control (*** *p*-value ≤ 0.001, **** *p*-value ≤ 0.0001). Statistical differences (*p* ≤ 0.05) between the samples are identified with different letters. MW RW—MW-pretreated red wine lees extract; MW P—MW-pretreated Port wine lees extract; GM—grape marc conventional extract; MW80 GM—MW-pretreated grape marc extract (max. temp. 80 °C).

In order to better understand the potential protective effects of the extracts towards keratinocytes and fibroblasts, further studies were performed using an oxidative stress inducer, TBHP, which is a more stable alkyl derivative of H_2_O_2_ that can initiate radical reactions, leading to damage of biomolecules. The effects of the extracts on TBHP-induced ROS was assessed in two different conditions: Pre-incubation of cells with the extracts prior to the addition of the stressor, and the co-incubation of the extracts with the stressor. Pre-incubation may reflect a preventive action, whereas co-incubation is more representative of a possible therapeutic approach. Several concentrations of the extracts were tested, and in both experiments a dose-dependent effect was observed (Figures S4 and S5). The results for the common concentration amongst the extracts (0.25 mg extract/mL) are presented in Figure 3. When comparing the two approaches, it is clear that the prevention of ROS formation is much more effective when the extracts are co-incubated with the stressor, with all the samples presenting a significant (*p* ≤ 0.0001) decrease of ROS in contrast with the untreated control (Figure 3C,D). These findings suggest that there must be compounds in the extracts that are not capable of permeating the cell membrane, and therefore their antioxidant properties can only be noticed when the induction of oxidative stress occurs in the presence of the extracts. In fact, certain phenolics may not be within the specific structural limitations required for membrane permeation, and therefore do not reach intracellular space. These observations are consistent with those reported elsewhere, in which *Opuntia ficus-indica* extracts [50] and traditional Portuguese cherry extracts [51] reveal a stronger antioxidant activity in co-incubation conditions rather than in the pre-incubation approach. The high molecular weight polyhydroxylated phenolics, such as certain flavonoids and their respective conjugates, might be among the compounds responsible for the distinction between the pre-incubation and co-incubation results, due to their difficulty permeating membranes [52]. Indeed, the inhibition of TBHP-induced ROS correlates better with TPC, TAC and the relative amount of flavonols in the case of co-incubation (R^2^ ≥ 0.92 in both cell lines) rather than in pre-incubation (R^2^ ≤ 0.63 in both cell lines). This makes sense, since in the co-incubation approach, all the compounds are present in the moment of stress induction, even those not able to permeate cells.

**Figure 4:**
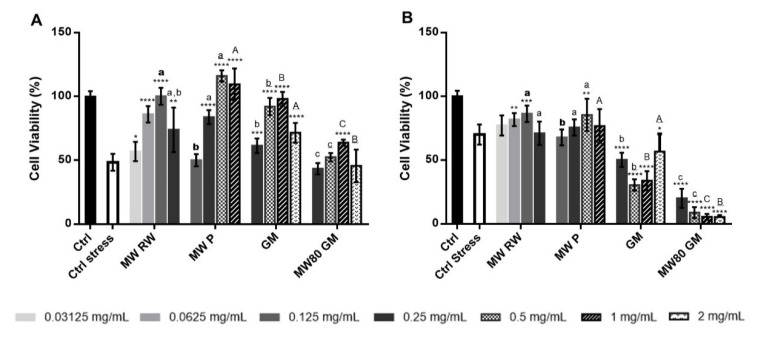
Pre-incubation of (**A**) HaCaT and (**B**) HFF with the extracts for 24 h – influence on cell viability upon TBHP-induced stress. The symbol * indicates significance relative to the control (* *p*-value≤ 0.05, ** *p*-value ≤ 0.01, *** *p*-value ≤ 0.001, **** *p*-value ≤ 0.0001). The same concentrations of different extracts were compared (bold lowercase letters for 0.125 mg/mL; regular lowercase letters for 0.25 mg/mL; underlined lowercase letters for 0.5 mg/mL; regular uppercase letters for 1 mg/mL; underlined uppercase letters for 2 mg/mL). Statistically different results (*p*-value ≤ 0.05) are identified with different letters. MW RW—MW-pretreated red wine lees extract; MW P—MW-pretreated Port wine lees extract; GM—grape marc conventional extract; MW80 GM—MW-pretreated grape marc extract (max. temp. 80 °C).

The assessment of the protective effects of natural extracts against oxidative damage caused by an oxidative stress inducer is commonly performed in keratinocytes and fibroblasts [53,54]. In this approach, the cells were pre-incubated with the extracts for 24 h, and then a cytotoxic level of oxidative stress was induced with TBHP for 1 h. Finally, the MTS assay was performed, and the cell viability of the treated cells was compared to an untreated control where stress was also induced (Ctrl stress). In Figure 4, particularly in the case of HaCaT (Figure 4A), a biphasic dose-response can be seen, in which the extracts lead to beneficial effects until a specific concentration is reached, and then cytotoxicity emerges. This specific response is referred to as hormesis, and results from an adaptive response of an organism upon disruption in homeostasis caused by low doses of an exogenous factor, whereas at high doses the toxic effect prevails [55]. Although phenolics are renowned antioxidants, they can also have pro-oxidant effects in certain conditions, often presenting hormetic responses. For instance, although phenolics can chelate transition metal ions, in some cases a redox reaction takes place instead, yielding reactive metal ions even more prone to participate in the Fenton chemistry, as well as phenolic intermediates (phenoxyl radicals) with pro-oxidant properties [56]. However, in HFF (Figure 4B), this effect is not as evident as in HaCaT. In fact, all concentrations of the grape marc extracts seem to have potentiated the cytotoxic effect caused by TBHP, possibly because the amount of TBHP-induced ROS led to an exhaustion of the antioxidant capacity of the phenolics present in the extracts, triggering pro-oxidant effects that might have caused a further decrease in cell viability. Nonetheless, at a concentration of 0.25 mg extract/mL in both cell lines, the wine lees extracts either prevented TBHP-induced cytotoxicity, or did not present any differences relative to the control, yielding overall better results than the grape marc extracts.

## 4. Conclusions

As concluding remarks, the red wine lees extracts presented the highest phenolic and anthocyanin contents, leading to distinguishably better results than all the other tested extracts in terms of antioxidant activity, as measured by ORAC, HOSC and HORAC, tyrosinase, elastase and MMP-1 inhibitory capacity, and the protection of human skin cells (keratinocytes and fibroblasts) against oxidative stress. Moreover, the MW-pretreatment of raw materials seems to contribute to phenolic and anthocyanin richness of the extracts. Nonetheless, winemaking waste streams, in particular wine lees, are indeed valuable sources of natural bioactives with the potential for application in cosmeceutical products with antioxidant, skin whitening and anti-ageing effects.

## Figures and Tables

**Table 1:** Phytochemical composition and antioxidant activity of the extracts. The results identified with different letters (a to f) in the same column are statistically different (*p*-value ≤ 0.05).

		Phytochemical Composition		Antioxidant Activity		
	Extract	TPC (mg GAE/g Extract)	TAC (mg malv-3-O-gl/g Extract)	ORAC (µmol TE/g Extract)	HOSC (µmol TE/g Extract)	HORAC (µmol CAE/g Extract)
Wine lees	RW	237.4 ± 7.7 ^a^	28.6 ± 2.4 ^a^	3167 ± 189 ^a^	3680 ± 163 ^a^	1932 ± 130 ^a^
	MW RW	266.0 ± 5.6 ^b^	29.5 ± 2.3 ^a^	3500 ± 223 ^a^	4776 ± 268 ^b^	2625 ± 135 ^b^
	P	64.0 ± 2.7 ^c^	6.1 ± 0.7 ^b^	451 ± 26 ^b^	837 ± 49 ^c^	458 ± 29 ^c^
	MW P	114.5 ± 4.7 ^d^	11.5 ± 1.0 ^c^	716 ± 41 ^c^	1285 ± 95 ^d^	776 ± 49 ^d^
Grape marc	GM	83.9 ± 2.0 ^e^	1.7 ± 0.1 ^d^	481 ± 30 ^b^	746 ± 49 ^c^	305 ± 28 ^e^
	MW80 GM	45.9 ± 1.5 ^f^	2.7 ± 0.3 ^e^	448 ± 31 ^b^	441 ± 34 ^e^	198 ± 19 ^f^

RW—red wine lees conventional extract; MW RW—MW-pretreated red wine lees extract; P—Port wine lees conventional extract; MW P—MW-pretreated Port wine lees extract; GM—grape marc conventional extract; MW80 GM—MW-pretreated grape marc extract (max. temp. 80 °C).

**Table 2:** The putative identification of phenolic compounds present in grape marc, red wine lees and Port wine lees, as obtained by HPLC-DAD-MS/MS. The retention times, *m*/*z* values and respective fragments, as well as the phenolic subclass, are presented.

Retention Time (min)	*m/z*	Ionic Species	Fragment Ions	Putative Identification	Phenolic Subclass	Grape Marc	Red Wine Lees	Port Wine Lees
23.2	169	[M−H]^−^	125	Gallic acid	Phenolic acid	✓	✓	✓
26.6	616	[M−H]^−^	466, 307, 272,167, 134	2-S-glutathionylcaftaric acid	Phenolic acid	✓	✓	✓
27.7	577	[M−H]^−^	289	Procyanidin dimer	Flavanol	✓	✓	✓
28.4	311	[M−H]^−^	179, 149, 135	Caftaric acid	Phenolic acid	✓	✓	✓
28.9	865	[M−H]^−^	577, 289	Procyanidin trimer	Flavanol	✓	✓	✓
29.8	289	[M−H]^−^	245	Catechin	Flavanol	✓	✓	✓
30.1	465	[M−H]^+^	303	Delphinidin-3-O-glucoside	Anthocyanin	✓	✓	✓
30.2	577	[M−H]^−^	289, 175, 129	Procyanidin dimer	Flavanol	✓	✓	✓
30.2	463	[M−H]^−^	300	Quercetin-3-O-glucoside	Flavonol	✓	✓	✓
31.1	295	[M−H]^−^	163, 149, 119	Coutaric acid	Phenolic acid	✓	✓	✓
31.5	289	[M−H]^−^	245	Epicatechin	Flavanol	✓	✓	✓
31.9	449	[M−H]^+^	287	Cyanidin-3-O-glucoside	Anthocyanin	✓	✓	✓
32.1	561	[M−H]^+^	399	Vitisin A	Pyranoanthocyanin	✓	✓	✓
32.2	479	[M−H]^+^	317	Petunidin-3-O-glucoside	Anthocyanin	✓	✓	✓
33.0	479	[M−H]^−^	316	Myricetin-3-O-glucoside	Flavonol	✓	✓	✓
34.0	493	[M−H]^+^	331	Malvidin-3-O-glucoside	Anthocyanin	✓	✓	✓
34.1	463	[M−H]^+^	301	Peonidin-3-O-glucoside	Anthocyanin	✓	✓	✓
34.9	477	[M−H]^−^	301	Quercetin-3-O-glucuronide	Flavonol	✓	✓	✓
34.9	507	[M−H]^+^	303	Delphinidin-3-O-6”-*p*-acetylglucoside	Anthocyanin	✓	✓	✓
36.4	707	[M−H]^+^	399	10-carboxypyranomalvidin-3-6”-*p*-coumaroylglucoside	Pyranoanthocyanin	✓	✓	✓
36.9	521	[M−H]^+^	317	Petunidin-3-O-6”-*p*-acetylglucoside	Anthocyanin	✗	✓	✓
36.9	507	[M−H]^−^	345	Syringetin-3-O-glucoside	Flavonol	✓	✓	✓
37.1	491	[M−H]^+^	287	Cyanidin-3-O-6”-*p*-acetylglucoside	Anthocyanin	✗	✗	✓
39.6	317	[M−H]^−^	179, 151, 137	Myricetin	Flavonol	✓	✓	✓
39.2	535	[M−H]^+^	331	Malvidin-3-O-6”-*p*-acetylglucoside	Anthocyanin	✗	✓	✓
39.5	505	[M−H]^+^	301	Peonidin-3-O-6”-*p*-acetylglucoside	Anthocyanin	✗	✓	✓
39.8	611	[M−H]^+^	303	Delphinidin-3-O-6”-*p*-coumaroylglucoside	Anthocyanin	✓	✓	✓
41.9	595	[M−H]^+^	287	Cyanidin-3-O-6”-*p*-coumaroylglucoside	Anthocyanin	✓	✓	✓
41.9	625	[M−H]^+^	317	Petunidin-3-O-6”-*p*-coumaroylglucoside	Anthocyanin	✓	✓	✓
43.9	301	[M−H]^−^	179,151, 121, 107	Quercetin	Flavonol	✓	✓	✓
43.8	639	[M−H]^+^	331	Malvidin-3-O-6”-*p*-coumaroylglucoside	Anthocyanin	✓	✓	✓
44.3	609	[M−H]^+^	301	Peonidin-3-O-6”-*p*-coumaroylglucoside	Anthocyanin	✓	✓	✓
48.2	285	[M−H]^−^	125	Kaempferol	Flavonol	✓	✓	✓
49.2	315	[M−H]^−^	300, 247, 215, 165, 141	Rhamnetin	Flavonol	✗	✓	✓

**Table 3:** The IC_50_ values of extracts towards tyrosinase, elastase and MMP-1. The results identified with different letters (a to d) in the same column are statistically different (*p*-value ≤ 0.05).

		Anti-hyperpigmentation Activity	Anti-ageing Activity	
	Extract	IC_50_ Tyrosinase (mg Extract/mL)	IC_50_ Elastase (mg Extract/mL)	IC_50_ MMP-1 (mg Extract/mL)
Wine lees	RW	0.20 ± 0.01 ^a^	0.17 ± 0.01 ^a^	0.22 ± 0.01 ^a^
	MW RW	0.14 ± 0.01 ^a^	0.11 ± 0.00 ^a^	0.21 ± 0.01 ^a^
	P	1.06 ± 0.07 ^b^	1.92 ± 0.09 ^b^	1.25 ± 0.03 ^b^
	MW P	0.62 ± 0.04 ^a,b^	0.83 ± 0.04 ^c^	0.65 ± 0.03 ^a,c^
Grape marc	GM	4.03 ± 0.14 ^c^	0.87 ± 0.03 ^c^	1.08 ± 0.08 ^b,c^
	MW80 GM	4.00 ± 0.14 ^c^	3.43 ± 0.11 ^d^	1.16 ± 0.06 ^b^

RW—red wine lees conventional extract; MW RW—MW-pretreated red wine lees extract; P—Port wine lees conventional extract; MW P—MW-pretreated Port wine lees extract; GM—grape marc conventional extract; MW80 GM—MW-pretreated grape marc extract (max. temp. 80 °C).

**Table 4:** Summary of the results obtained by the extracts in chemical and enzymatic assays, in terms of phytochemical composition, antioxidant, anti-hyperpigmentation and anti-ageing activities. For each assay, the extracts were rated based on their percentage relative to the mean. Phytochemical composition and antioxidant activity: - for 0–50%; + for 50–100%; ++ for 100–150%; +++ for 150–200%; ++++ for 200–250%; +++++ for >250%. Enzymatic assays: +++++ for 0–10%; ++++ for 10–25%; +++ for 25–50%; ++ for 50–100%; + for 100–200%; - for >200%.

		Phytochemical Composition		Antioxidant Activity			Anti-hyperpigmentation Activity	Anti-ageing Activity	
	Extract	TPC	TAC	ORAC	HOSC	HORAC	Tyrosinase	Elastase	MMP-1
**Wine lees**	**RW**	+++	++++	++++	++++	++++	+++++	++++	+++
	**MW RW**	++++	+++++	+++++	+++++	+++++	+++++	+++++	++++
	**P**	+	+	-	-	+	+++	+	+
	**MW P**	+	+	+	+	++	+++	++	++
**Grape marc**	**GM**	+	-	-	-	-	+	++	+
	**MW80 GM**	-	-	-	-	-	+	-	+

RW—red wine lees conventional extract; MW RW—MW-pretreated red wine lees extract; P—Port wine lees conventional extract; MW P—MW-pretreated Port wine lees extract; GM—grape marc conventional extract; MW80 GM—MW-pretreated grape marc extract (max. temp. 80 °C).

## Supplementary Materials

The following are available online at https://www.mdpi.com/2076-3921/8/9/355/s1, Figure S1: Cytotoxicity screening of the chosen extracts (24 and 48 h of incubation) in HaCaT, Figure S2: Cytotoxicity screening of the chosen extracts (24 and 48 h of incubation) in HFF, Figure S3: Pre-incubation of the cells with four concentrations of each extract for 1 h—effect on endogenous ROS, Figure S4: Pre-incubation of the cells with four concentrations of each extract for 1 h—effect on TBHP-induced ROS, Figure S5: Co-incubation of the cells with TBHP and four concentrations of each extract for 1 h. (A) HaCaT; (B) HFF.
