# Supplementary material for: Polyphenol-Rich Extracts Obtained from Winemaking Waste Streams as Natural Ingredients with Cosmeceutical Potential
Source: Antioxidants (Basel). 2019 Sep 1;8(9):355. doi: 10.3390/antiox8090355 (PMC6770854; doi:10.3390/antiox8090355)
Supplement: Supplementary file 1 [file antioxidants-08-00355-s001.pdf]

Supplementary material

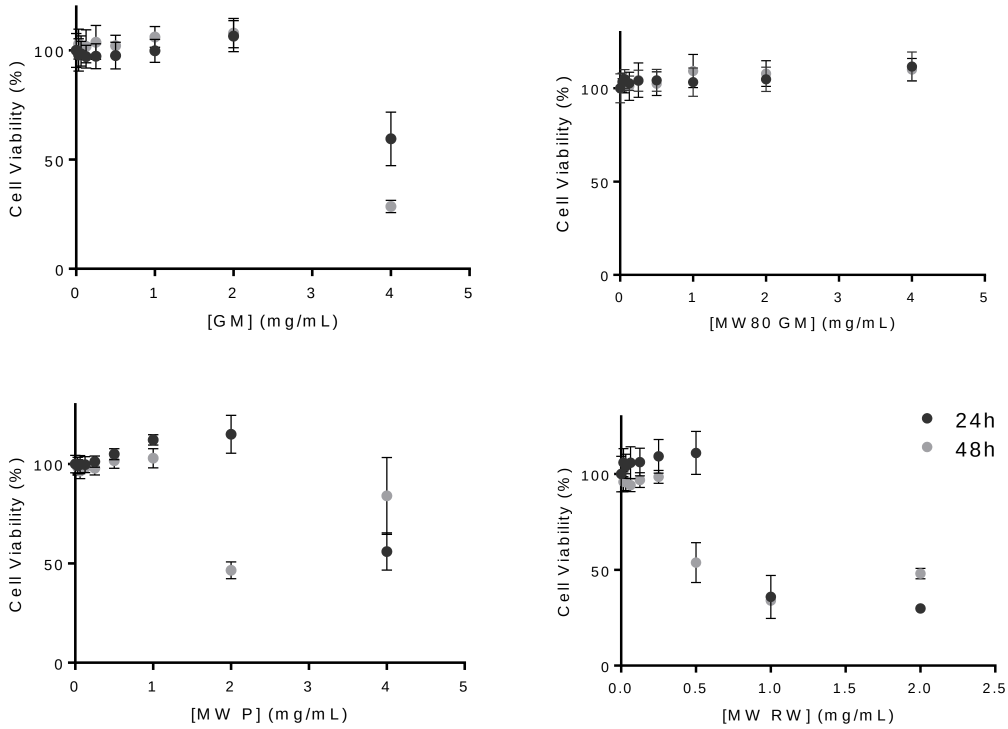

Figure S1. Cytotoxicity screening of the chosen extracts (24 and 48 hours of incubation) in HaCaT. Results were obtained from three independent experiments. GM – grape marc conventional extract; MW80 GM – MW-pretreated grape marc extract (max. temp. 80ºC); MW P – MW-pretreated Port wine lees extract; MW RW – MW-pretreated red wine lees extract. Results were obtained from three independent experiments.

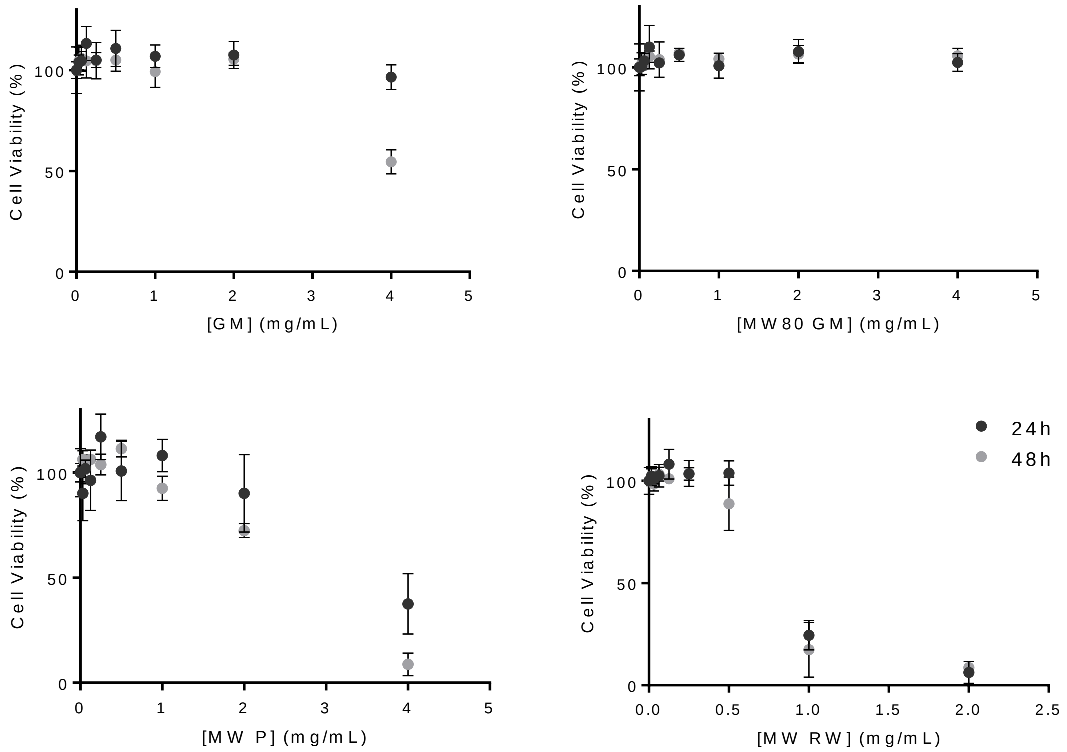

Figure S2. Cytotoxicity screening of the chosen extracts (24 and 48 hours of incubation) in HFF. Results were obtained from three independent experiments. GM – grape marc conventional extract; MW80 GM – MW-pretreated grape marc extract (max. temp. 80ºC); MW P – MW-pretreated Port wine lees extract; MW RW – MW-pretreated red wine lees extract. Results were obtained from three independent experiments.

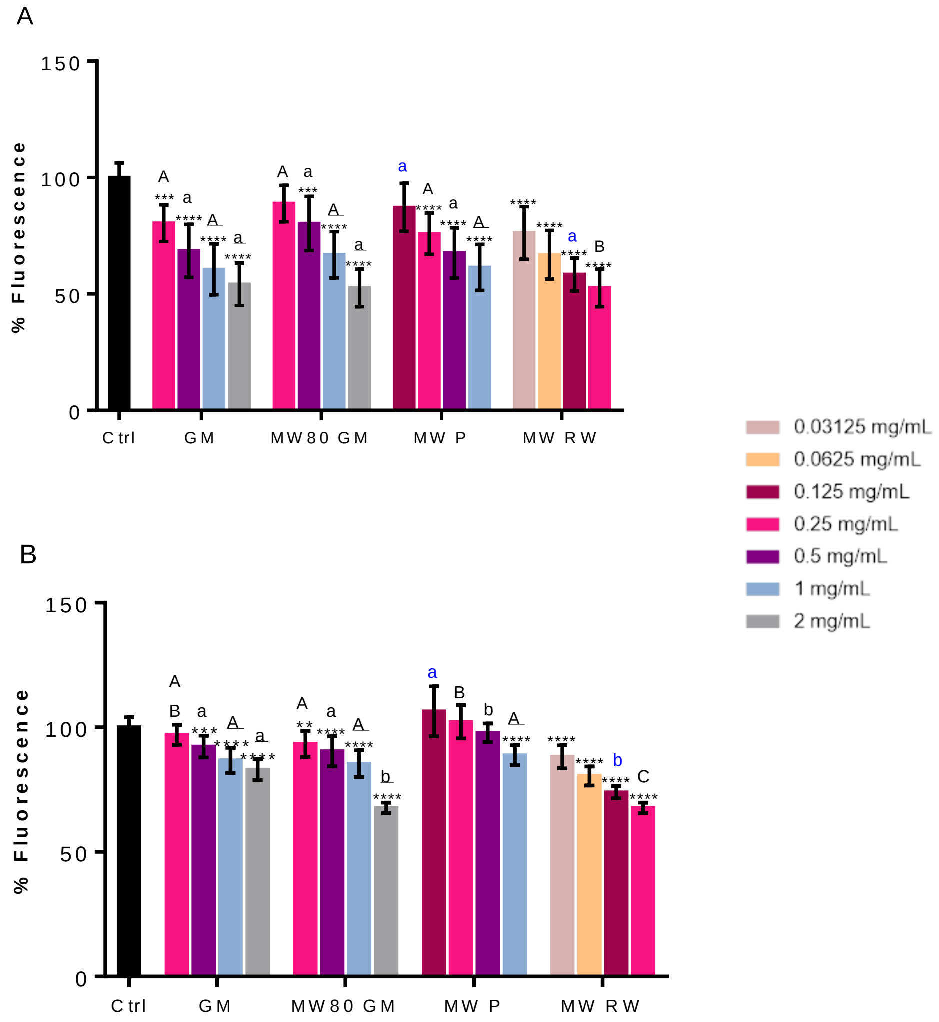

Figure S3. Pre-incubation of cells with four concentrations of each extract for 1h – effect on endogenous ROS. (A) HaCaT; (B) HFF. The symbol * indicates significance relative to the control (*p-value≤0.05, **p-value≤0.01, ***p-value≤0.001, ****p-value≤0.0001). The same concentrations of different extracts were compared (blue lowercase letters for 0.125 mg/mL; uppercase letters for 0.25 mg/mL; black lowercase letters for 0.5 mg/mL; underlined uppercase letters for 1 mg/mL; underlined lowercase letters for 2 mg/mL); statistically different results (p-value≤0.5) are identified with different letters. GM – grape marc conventional extract; MW80 GM – MW-pretreated grape marc extract (max. temp. 80ºC); MW P – MW-pretreated Port wine lees extract; MW RW – MW-pretreated red wine lees extract. Results were obtained from three independent experiments.

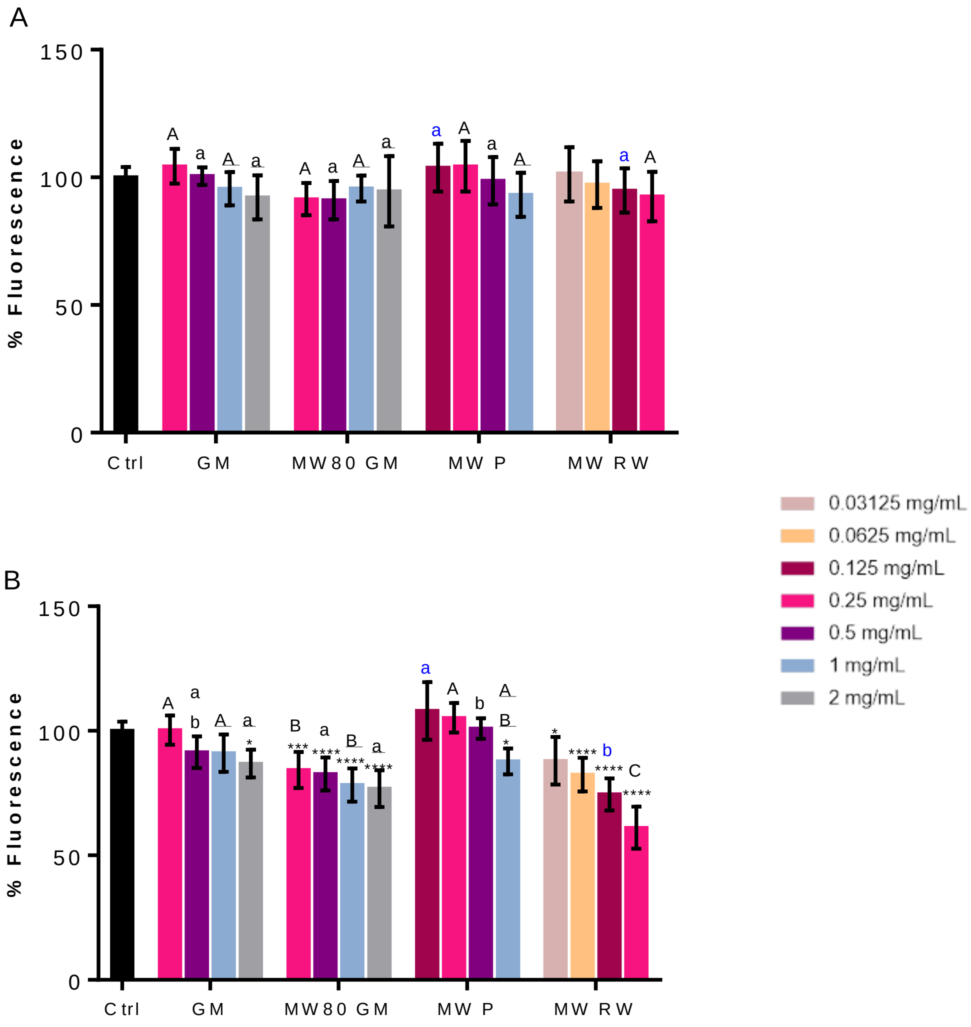

Figure S4. Pre-incubation of cells with four concentrations of each extract for 1h – effect on TBHP-induced ROS. (A) HaCaT; (B) HFF. The symbol * indicates significance relative to the control (*p-value≤0.05, **p-value≤0.01, ***p-value≤0.001, ****p-value≤0.0001). The same concentrations of different extracts were compared (blue lowercase letters for 0.125 mg/mL; uppercase letters for 0.25 mg/mL; black lowercase letters for 0.5 mg/mL; underlined uppercase letters for 1 mg/mL; underlined lowercase letters for 2 mg/mL); statistically different results (p-value≤0.5) are identified with different letters. GM – grape marc conventional extract; MW80 GM – MW-pretreated grape marc extract (max. temp. 80ºC); MW P – MW-pretreated Port wine lees extract; MW RW – MW-pretreated red wine lees extract. Results were obtained from three independent experiments.

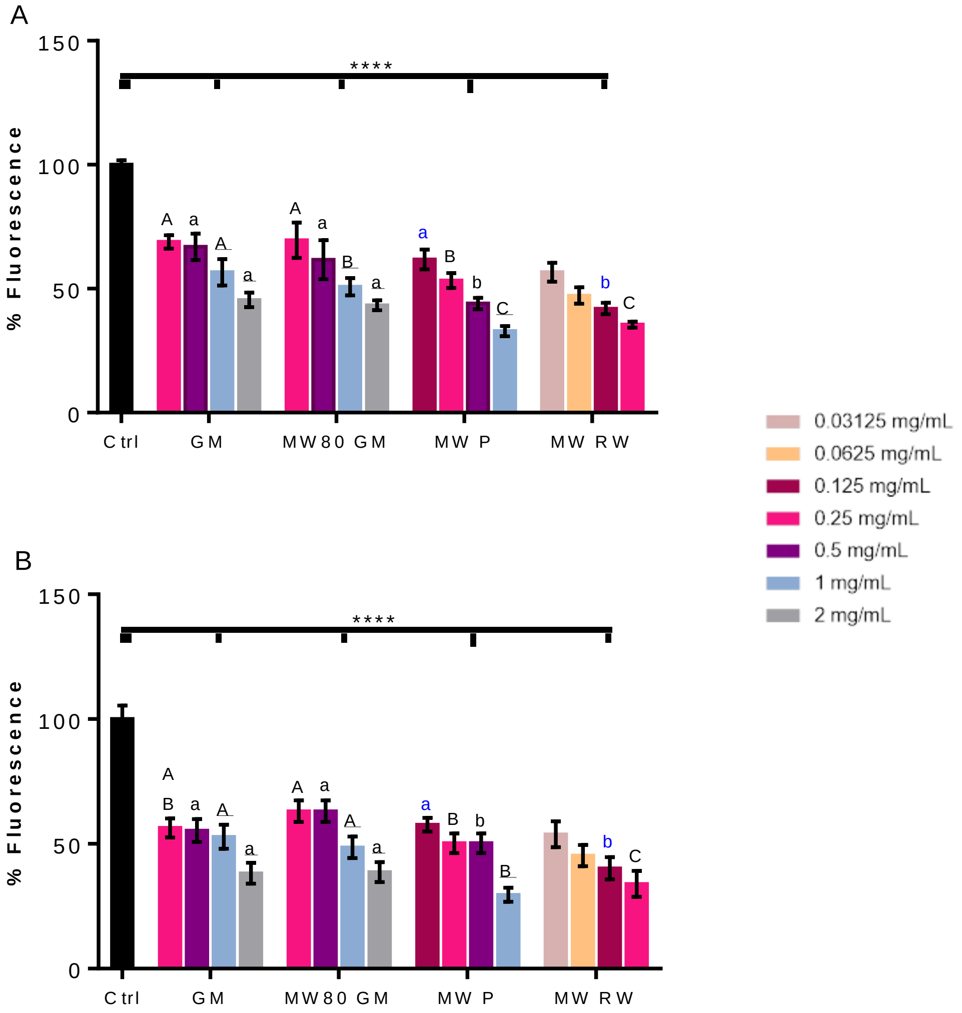

Figure S5. Co-incubation of cells with TBHP and four concentrations of each extract for 1h. (A) HaCaT; (B) HFF. The symbol * indicates significance relative to the control (*p-value≤0.05, **p-value≤0.01, ***p-value≤0.001, ****p-value≤0.0001). The same concentrations of different extracts were compared (blue lowercase letters for 0.125 mg/mL; uppercase letters for 0.25 mg/mL; black lowercase letters for 0.5 mg/mL; underlined uppercase letters for 1 mg/mL; underlined lowercase letters for 2 mg/mL); statistically different results (p-value≤0.5) are identified with different letters. GM – grape marc conventional extract; MW80 GM – MW-pretreated grape marc extract (max. temp. 80ºC); MW P – MW-pretreated Port wine lees extract; MW RW – MW-pretreated red wine lees extract. Results were obtained from three independent experiments.
